# Tumor acidity: From hallmark of cancer to target of treatment

**DOI:** 10.3389/fonc.2022.979154

**Published:** 2022-08-29

**Authors:** Alexey Bogdanov, Andrey Bogdanov, Viacheslav Chubenko, Nikita Volkov, Fedor Moiseenko, Vladimir Moiseyenko

**Affiliations:** Saint Petersburg Clinical Research and Practical Center of Specialized Types of Medical Care (Oncological), Saint Petersburg, Russia

**Keywords:** cancer, metabolism, acidity, hallmark, treatment target

## Abstract

Tumor acidity is one of the cancer hallmarks and is associated with metabolic reprogramming and the use of glycolysis, which results in a high intracellular lactic acid concentration. Cancer cells avoid acid stress major by the activation and expression of proton and lactate transporters and exchangers and have an inverted pH gradient (extracellular and intracellular pHs are acid and alkaline, respectively). The shift in the tumor acid–base balance promotes proliferation, apoptosis avoidance, invasiveness, metastatic potential, aggressiveness, immune evasion, and treatment resistance. For example, weak-base chemotherapeutic agents may have a substantially reduced cellular uptake capacity due to “ion trapping”. Lactic acid negatively affects the functions of activated effector T cells, stimulates regulatory T cells, and promotes them to express programmed cell death receptor 1. On the other hand, the inversion of pH gradient could be a cancer weakness that will allow the development of new promising therapies, such as tumor-targeted pH-sensitive antibodies and pH-responsible nanoparticle conjugates with anticancer drugs. The regulation of tumor pH levels by pharmacological inhibition of pH-responsible proteins (monocarboxylate transporters, H^+^-ATPase, etc.) and lactate dehydrogenase A is also a promising anticancer strategy. Another idea is the oral or parenteral use of buffer systems, such as sodium bicarbonate, to neutralize tumor acidity. Buffering therapy does not counteract standard treatment methods and can be used in combination to increase effectiveness. However, the mechanisms of the anticancer effect of buffering therapy are still unclear, and more research is needed. We have attempted to summarize the basic knowledge about tumor acidity.

## Introduction

Cancer cells have an inverted pH gradient: extracellular and intracellular pHs (pHe, pHi) are acid and alkaline, respectively (1). The acid shift in the tumor microenvironment (TME) is closely associated with hypoxia (2) but, more specifically, with highly activated glycolysis in tumor cells. Even in normoxia, about 80% of all malignant tumors use aerobic glycolysis, described as the Warburg effect (3), which is an integral part of metabolic reprogramming and sustaining biosynthetic pathways in cancer cells (4).

**Figure 1:**
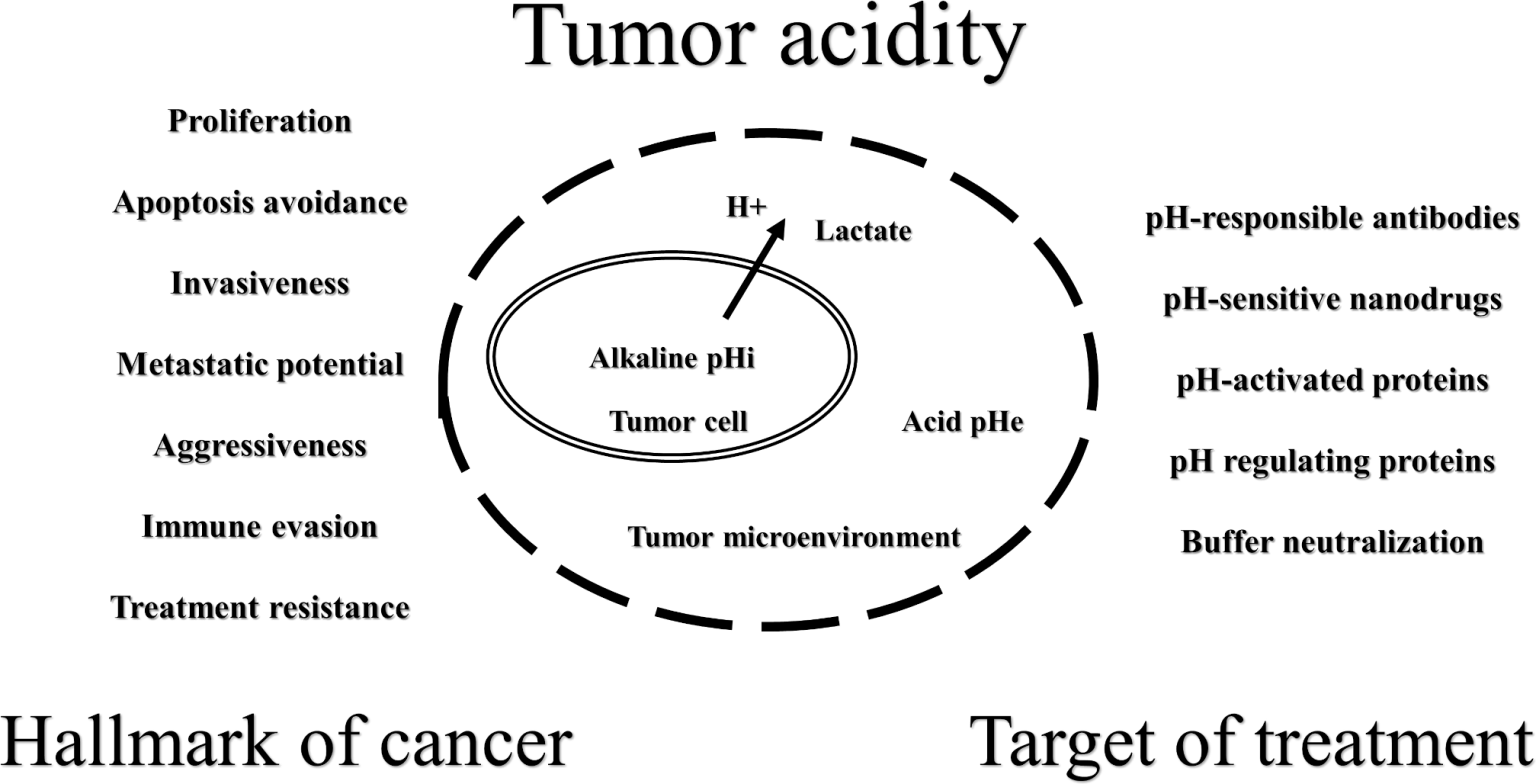
Schematic representation of tumor acidity properties.

According to the present knowledge, the shift in the tumor acid-base balance promotes proliferation, apoptosis avoidance, invasiveness, metastatic potential, aggressiveness, immune evasion, and treatment resistance (5–8). On the other hand, inversion of the pH gradient in tumors could be a weakness that will allow for the development of new promising therapies (**Figure 1**). It is possible to create acid stress inside cancer cells by inhibiting proton release systems or by using drugs that decrease mitochondrial activity to increase lactate production (5, 9, 10). The acidity of the TME could be used for the drug delivery of cytotoxic agents and/or carriers that are more active and/or change physicochemical properties under such conditions (11–13). It is very attractive to increase the pHe by a combination of an alkaline diet and bicarbonate therapy (14–16) or by direct local isolated perfusion of the tumor with bicarbonate solutions (17, 18).

Obviously, the altered acid-base state of the tumor affects every stage of cancer development, from dysplasia to metastatic disease (1, 2). In this mini-review, we have attempted to summarize the basic knowledge about tumor acidity from hallmark of cancer to target of treatment.

## Tumor acidity as a hallmark of cancer

One of the causes of tumor heterogeneity is altered tumor vasculature, which leads to different perfusion of nutrients and oxygen and to the accumulation of acidic metabolites (19, 20). Due to the reprogramming of metabolism in such conditions and the use of glycolysis as a major source of ATP production, tumor cells have an acidic pHe (6.4-7.1) and an alkaline pHi (7.1-7.8). For normal tissues, the pHe is around 7.4, and the pHi is around 7.2 (2, 21). Large amounts of lactate produced during glycolysis result in a significant increase in the intracellular proton (H+) concentration. It should be noted that glutaminolysis is another way for ATP production and an additional source of lactate and H+ in cancer cells (21–24). In addition, glutamine uptake and metabolism in oxidative cancer cells can be promoted by lactate (25). However, even in the presence of oxygen, glucose is almost completely converted into lactate. At the same time, glutamine is not fully respired, but it is rather fermented into lactate or pyruvate. Increased glutamine flux can enhance aerobic glycolysis and make it optimal for tumor proliferation (22, 26).

As acid stress triggers apoptosis (27), cancer cells use several ways to evade it (28). Activation and expression of H+ (and lactate) transporters and exchangers are the main mechanisms of tumor cell adaptation to intracellular acidification and of the inverted pH gradient phenomenon (29–32). It should be noted that not only H+ ejection systems lead to an increase in the pHi, but also a reduction of CO_2_ by decreased activity of the tricarboxylic acid cycle (TCA) and oxidative phosphorylation (OXPHOS) (1, 10). Carbonic anhydrases (CAs) additionally support the pH regulation of cancer cells by catalyzing the reversible hydration of CO_2_ to HCO_3_− and H+ (32).

Acidosis of the TME is an essential stage associated with high rates of tumor cell proliferation (33). Numerous studies have shown a role for tumor acidity in acquiring aggressive cancer characteristics, so it is recognized as a hallmark of cancer (21, 31, 34–36). For example, melanoma cells exposed to acidosis are characterized by a high invasive potential, high resistance to apoptosis and drug therapy, fixed independent growth, and a phenotype of epithelial to mesenchymal transition (37). Under growth factor limitations, alkaline pHi favors cancer cells survival (38). The acid adaptation of tumor cells leads to a gene expression response that correlates with human cancer tissue gene expression profiles and survival (39). Acidic TME improves the activity of regulatory T-cells and inhibits effector T-cells (40). In view of the foregoing, acidic TME could serve as an incubator that represses overabundant proliferation and cultures cells with a restricted growth rate but with strong proliferative potential (41). Clinicians should consider tumor acidity when diagnosing and determining optimal treatment, as it is also connected with poor cancer patients prognosis (39).

A wide range of non-invasive and minimally invasive imaging modalities have been studied preclinically for tumor pH monitoring, including magnetic resonance imaging (MRI) and spectroscopy, positron emission tomography, electron paramagnetic resonance, and optical and photoacoustic imaging (42). To date, among the methods used, MRI appears to be the most promising, particularly chemical exchange saturation transfer (CEST) MRI, which has good *in vivo* sensitivity for assessing tumor acidosis and changes in pH after therapeutic treatment, with a high spatial resolution to determine the heterogeneity of extracellular acidification. For example, CEST MRI has been used successfully to map tumor pH in a rabbit liver cancer model (43). In another study, tumor acidosis assessed by CEST MRI revealed the metastatic potential of breast cancer in mice (44). Translating the results of preclinical studies into clinical trials is only beginning to yield significant results. CEST MRI shows good results for measuring pH in ovarian cancer patients (45). In addition, CEST MRI has recently been shown to differentiate between benign and malignant liver tumors in patients (46). However, it is still difficult to routinely measure the pH of tumors in the clinic. In addition to direct measurements, tumor acidity can be assessed indirectly by determining the concentrations of bicarbonate (47) and lactate ions in the blood and using biopsy data (48). However, each clinical situation requires an individual approach.

## Tumor acidity and tumor resistance

Cancer cell survival strategies in acidic TME promote resistance to radiation and chemotherapy. Radioresistance is closely related to hypoxia. Available clinical data show that the presence of large hypoxia areas in solid tumors is associated with a poor prognosis in cancer patients after radiotherapy (49). The cytotoxic effects of ionizing radiation are mainly due to damage to genomic DNA as a result of the indirect action of generated free radicals (50). Molecular oxygen must be present during irradiation, which is insufficient under hypoxic conditions. Hypoxia also prevents DNA repair and leads to the inhibition of the G1/S cell cycle checkpoint, an increase in DNA errors, and an increase in chromosomal instability. At the same time, the alkaline pHi of tumor cells prevents mitotic arrest initiated by activated checkpoints during DNA damage (21, 51). Thus, the inversion of the pH gradient of the tumor is a “partner” of hypoxia in creating conditions for radioresistance, and clinicians should consider acidic TME in the planning of radiation therapy.

Acidic TME itself can lead to chemoresistance due to ongoing physicochemical changes in the structure and charge of drugs. Weak-base chemotherapeutic agents, such as vincristine, mitoxantrone, doxorubicin, vinblastine, and paclitaxel, may have substantially reduced cellular uptake capacity due to neutralization or protonation [“ion trapping” (52)]. Therefore, the cytotoxic effects of these drugs may be reduced, resulting in a stable tumor phenotype. Interestingly, reversing the pH gradient may increase the intracellular concentrations of some weak-acid drugs, including cyclophosphamide and chlorambucil (53–56). Acidic TME induces p-glycoprotein (multiple drug resistance (MDR) protein) activity by promoting p38 mitogen-activated protein kinase (57–59). Tumor acidosis induces the expression of the transcription factor SOX2 by inhibiting vitamin D receptor-mediated transcription, which also results in drug resistance (60). Oxidation-induced lactic acidosis increases resistance to uprosertib, a serine/threonine protein kinase inhibitor, in colon cancer cells (61). To obtain the maximum effect of chemotherapy, the acidity of the TME must be considered.

Current knowledge strongly suggests that acidic TME inhibits the antitumor immune response, although the complication of experimentally measuring tumor acid-base status makes it difficult to obtain direct evidence (7, 62). For instance, a decrease in the pHe leads to a decrease in the activity and proliferation of T cells (63, 64). In an acidic environment, effector T cells require higher thresholds for full activation and co-stimulatory signals (e.g., CD28) and show increased negative regulatory signaling through upregulation of interferon gamma receptor 2 (IFN-γR2) and cytotoxic T cell-associated protein 4 (CTLA-4) (64). Acidic extracellular conditions reduce the expression of T-lymphocyte receptor components (65). Since the movement of lactate between the cytosol and the extracellular space depends on its concentration gradient, a high concentration of extracellular lactate in the TME prevents the export of lactate from T cells. This negatively affects the functions of activated T-lymphocytes dependent on glycolysis for ATP production (66). Notably, the functions of effector T- lymphocytes could be restored after normalization of pH (65–69), so the acidity does not have a cytotoxic effect. A significant effect of low acidity appears to be its negative effect on effector cytokines production by T cells, which is significantly reduced under acidic conditions (70–72). However, receptor interactions also play an important role. For example, in acidic TME, the V-domain Ig suppressor of T cell activation (VISTA), which is expressed by tumor-infiltrating myeloid suppressor cells, is activated and suppresses effector T cells (73). The inhibitory effect of acidic TME on dendritic cells is not related to the high concentration of H+, which actually stimulates antigen presentation (74). This inhibition can be explained by the accumulation of lactate, which modulates the dendritic cell phenotype and causes increased production of anti-inflammatory (e.g., IL-10) and decreased production of pro-inflammatory (e.g., IL-12) cytokines (75, 76). An acidic pHe and a high concentration of lactate together lead to a decrease in the activity of natural killers, including the depletion of interferon gamma (IFN-γ) and their ability to infiltrate the tumor (71, 77, 78). At the same time, for example, an acidic environment stimulates regulatory T cells (Tregs) activity by involving lactic acid in metabolism (79). In addition, lactic acid promotes Tregs’ expression of programmed cell death receptor 1 (PD-1) by absorption through monocarboxylate transporter 1 (MCT1). Thus, the PD-1 blockade activates PD-1-rich Tregs, resulting in treatment failure (80). Besides this, the acidity of the TME upregulates programmed death ligand 1 (PD-L1) in tumor cells (81).

It seems clear that the acidic conditions of the TME must be considered in monoclonal antibodies (mAbs) anticancer therapy. On the one hand, slightly acidic conditions are probably optimal for most mAbs (82), i.e., acidity in solid tumors may only slightly influence the deterioration of the therapeutic properties of mAbs. On the other hand, the possibility of the degradation of mAbs under such conditions cannot be excluded (7). For example, the rate of antibody Fc fragment oxidation and aggregation, which determines antibody-dependent cellular cytotoxicity (ADCC) and complement-dependent cytotoxicity (CDC), has been shown to increase with decreasing pH (83, 84). Despite the fact that cancer immunotherapy uses immune checkpoint-blocking mAbs that are specifically modified to eliminate interactions with Fc receptors, fatal changes in other parts of mAbs that determine their activity are also possible at low pH values. For example, the chemical degradation of aspartic acid induced by acidic pH in the complementarity-determining region (CDR) of a monoclonal antibody against the epidermal growth factor receptor (EGFR) causes a loss of antibody-binding activity (85). The high structural and physicochemical affinity of mAbs to their targets is a condition for achieving a therapeutic effect. In particular, histidine residues in interacting sites can increase pH-mediated dissociation due to protonation under acidic conditions, favoring electrostatic repulsion between rigid domains in protein–protein interaction (86). The low pHe can also greatly affect the bioavailability of therapeutic mAbs. At the same time, the “useful” side of acidic TME is the possibility of creating therapeutic pH-selective mAbs (87, 88).

## Tumor acidity as a target of treatment

Tumor-targeted pH-sensitive antibodies should be screened for low pH activity, and antibody engineering should not be limited to finding molecules with activity over a wide pH range (87). For example, despite the pH-independent affinity of CTLA-4 for ipilimumab, an analog was developed with up to a 50-fold affinity for CTLA-4 at pH 6.0 compared to pH 7.4 (89). A bispecific pH-responsive anticarcinoembryonic antigen-related cell adhesion molecule (CEACAM) 5 antibody that binds pH-independently to CEACAM6 was generated (88). Likewise, acidic TME allows for pH-activated molecular targets, such as VISTA. A combination of anti-VISTA mAb with anti-PD-L1 therapy demonstrated a significant survival benefit in tumor-bearing mice (90). Nanotechnologies also provide a good tool for creating pH-responsible anticancer drugs based on pH-responsible polymer nanomaterials, nanogels, etc. (91, 92). It has recently been summarized that several types of pH-sensitive nanoparticle conjugates with paclitaxel, doxorubicin, or others enhance drug delivery and potentiate anticancer effects in various experimental cancer cell lines (93).

Another approach to influencing tumor aggressiveness and/or therapeutic response is the regulation of tumor pH levels. First, since glycolysis is the main source of lactate and H+, would it be possible to reduce lactate production by limiting glucose? Also considering that hyperglycemia is known to be associated with reduced survival rates in some types of cancer (94–97), although this is still controversial, for example, in pancreatic (98–100) or colorectal cancers (101–103). Indeed, glucose restriction can reverse the Warburg effect and decrease lactate production *in vitro* (104). However, cancer cells can also use glycogenolysis, glycogen synthesis, and gluconeogenesis to compensate for glucose starvation (105–107). Many therapies targeting glucose metabolism (e.g., targeting glucose transporters, glycogen phosphorylase, glycogen synthase kinase 3β, hexokinase 2, glucose-6-phosphate isomerase, etc.) have been developed, but have not yet been successful in clinical trials (107). Furthermore, glycolysis is the main metabolic pathway of neutrophils, M1 macrophages, dendritic cells, naive T cells, effector T cells, etc. (108). For example, glucose-deficient TME limits the anaerobic glycolysis of tumor-infiltrating T cells and thus suppresses tumor-killing effects (109). Nutritional deficiencies in the TME, especially glucose, impair the metabolism of NK cells and their antitumor activity (110). It is important to note that human glucose levels may be reduced to very low levels without causing harm (111), and ketone bodies can be used for energy production with benefits for the organism (112, 113). For instance, a ketogenic diet improves the function of T cells (114, 115) and possibly creates an unfavorable metabolic environment for cancer cells (116, 117). However, ketone bodies utilization or formation may be a promoter for tumor cells proliferation and metastasis (118–121). Therefore, limiting glucose or its metabolism to reduce lactate production can have a completely ambiguous effect.

A more optimal way to reduce lactate production seems to be the inhibition of lactate dehydrogenase A (LDHA). This approach provides the simultaneous restriction of lactate synthesis from both glycolysis and glutaminolysis. Indeed, the inhibition of LDHA *in vivo* redirects pyruvate to support OXPHOS (122, 123). To date, a large number of LDHA inhibitors have been studied preclinically, but unfortunately, the clinical utility of such inhibitors may be limited due to nonselective toxicity or complex interactions with other cellular components. Optimization of existing compounds and continued search and development of new LDHA inhibitors will be reasonable strategies to obtain direct antitumor effects or enhance, for example, immunotherapy results (48, 124, 125). For example, since the effect of immunotherapy can be prevented by lactate (79, 80) and high LDH levels before treatment are correlated with a poor response to immunotherapy (126, 127), inhibition of LDHA can improve the efficacy of anti-PD-1 therapy (128).

Alternate modality to regulate tumor acidity is the pharmacological inhibition of proteins responsible for regulating pHi or mitochondrial activity (5, 9, 10). For example, inhibition of mitochondrial pyruvate transporter (MPC) works to block lactate utilization while preventing oxidative glucose metabolism (129). Blocking the monocarboxylate transporter 1 (MCT1) (used to import lactate as an energy source in oxidative cancer cells) with the specific MCT1 inhibitor AZD3965 prevents lactate consumption, increases its concentration in the TME, and has an antiproliferative effect (130–132). Conversely, the inhibition of MCT4 (expressed to remove lactate in glycolytic tumor cells) causes intracellular lactate accumulation, a decrease in pHi, but also reduces tumor growth *in vitro* and *in vivo* (132, 133). The cooperative use of MCT1/MCT4 inhibitors or nonspecific MCT inhibitors has good therapeutic potential (125, 132, 134, 135). Also of great importance to decrease pHi values is the pharmacological inhibition of the proton pump H^+^-ATPase (136), sodium-hydrogen antiporter 1 (NHE1) (137), and carbonic anhydrase IX (CAIX) (138). For example, according to the results of a phase III clinical trial (NCT01069081), intermittent use of a high dose of the proton pump inhibitor esomeprazole potentiates the effects of docetaxel and cisplatin chemotherapy in metastatic breast cancer without causing further toxicity (139). In a retrospective study, omeprazole was found to have a synergistic effect with chemoradiotherapy and to significantly reduce the risk of rectal cancer recurrence (140). Other ion exchangers and transporters are involved in tumor pH regulation, but their role in cancer progression remains unclear (2).

Another way to affect tumor acidity is the use of buffer systems, such as sodium bicarbonate. Preclinical and some clinical studies suggest that “direct” tumor deacidification may slow progression or improve therapeutic response (34). Oral administration of sodium bicarbonate can increase the efficacy of doxorubicin and mitoxantrone in model experiments (52, 55). Furthermore, peroral administration of sodium bicarbonate and other buffer solutions significantly reduced the invasion and metastasis of various experimental (including spontaneous) tumors in genetically modified animals but had no effect on the growth of primary tumors (141–146). Neutralization of tumor acidity improved the antitumor response to anti-CTLA-4 and PD-1 mAbs, as well as the adoptive transfer of T-lymphocytes in experiments using the B16 melanoma model and Panc02 pancreatic cancer in mice (69).

At the same time, the first three clinical trials of oral sodium bicarbonate (NCT01350583, NCT01198821, NCT01846429) to improve outcomes and reduce pain in pancreatic adenocarcinoma failed due to poor taste sensation and gastrointestinal disturbances, resulting in bad compliance (147). However, a recent clinical study successfully examined the effect of alkalinization therapy (an alkaline diet supplemented with oral sodium bicarbonate) in combination with chemotherapy on the survival of patients with advanced pancreatic cancer (UMIN 000035659). The median overall survival rate in patients whose urine pH became high (>7.0) after the start of therapy was significantly greater than in patients with low urine pH (≤7.0) (16.1 vs 4.7 months; p<0.05) (14). In another study (UMIN000043056), the combination of alkalinization therapy with intravenous vitamin C was also associated with favorable outcomes in patients with small cell lung cancer (SCLC) receiving chemotherapy. The median overall survival for the intervention group was 44.2 months vs. 17.7 months for the control group (15).

Parenteral administration of buffer systems to directly neutralize tumor acidity is also of great importance, but it must be done under the close supervision of medical personnel and can have some serious side effects (148). The use of nanoobjects to deliver buffers due to the enhanced permeability and retention effect (EPR) can overcome such limitations (149). For example, the administration of sodium bicarbonate-loaded liposomes in combination with subtherapeutic doses of doxorubicin in mice with triple-negative breast cancer resulted in a superior therapeutic response compared to drug administration alone (150). Performing an isolated infusion or perfusion of the tumor with buffer solutions is another option. In the ChiCTR-IOR-14005319 clinical study, the efficacies of transarterial chemoembolization (TACE) with or without local administration of 5% sodium bicarbonate solution in patients with large-focal hepatocellular carcinoma were compared. In the case of sodium bicarbonate, the objective response rate (ORR) was 100% vs. 44.4% in the case of conventional TACE in a nonrandomized cohort and 63.6% in a randomized study (151). In a preclinical study, it was found that intraperitoneal perfusion with 1% sodium bicarbonate solution significantly prolonged overall survival in mice with the ascitic form of Ehrlich’s adenocarcinoma (median survival, 24 vs. 17 days; p < 0.05) when compared to 0.9% sodium chloride solution (18). In another study, perfusion was performed with a 4% sodium bicarbonate solution of rat limbs with a Pliss lymphosarcoma graft. The median survival in the sodium bicarbonate group was 17 days, while in the nonperfused group and in the isotonic saline group it was 13 days (17).

The mechanisms of the anticancer effects of alkalization (buffering) therapy remain unclear. While the improved chemotherapeutic effect can be explained by “ion trapping” (53, 54), the antitumor, antimetastatic, and immunotherapy-enhancing effects of buffered therapy may be much more complex and have been studied predominantly as a phenomenon until now. Buffering of the TME can reduce the optimal conditions for enzymes involved in tumor invasion, such as cathepsins and matrix metalloproteases (MMPs) (152). Neutralization of acidity in the TME can result in a reduction of PD-L1 expression, which is increased at low pH through proton-sensing G protein-coupled receptors (81). Neutralization of lactic acid with sodium bicarbonate reactivates metabolically altered (in an acid environment) T cells, enabling extracellular lactate as an additional source for their energy production (153). More research is needed on the mechanisms of the effectiveness of sodium bicarbonate and other buffer solutions in cancer patients. Alkalization (buffering) therapy does not conflict with standard treatment methods but can be used in combination to increase effectiveness (154).

## Conclusion

Despite extensive studies on the acid-base status of malignant tumors over the past decades, the mechanisms of tumor adaptation to acidity, induction of invasion and metastasis, and the mechanisms leading to evasion of immune surveillance are still poorly understood. Further research in this direction is needed, including the development of approaches and drugs that directly or indirectly increase the pH of the TME for use in conjunction with chemotherapy, radiation therapy, and immunotherapy. However, it is clear that clinical options already exist to counteract tumor acidosis in patients. Additionally, the selectivity of acidosis in tumors versus healthy tissues holds promise for pH-activated or pH-targeted drugs, which are safer than traditional chemotherapy and are applicable to more cancers than many targeted drugs. Regardless of the complexity of the clinical assessment of the TME acidity, clinicians should consider acidosis in practice, and the continued development of methods for clinical assessment of tumor pH should allow for accurate diagnosis and selection of personalized treatment regimens.

## Author contributions

AlB and VM contributed to the conception of the mini-review. AlB and AnB wrote the first draft of the manuscript. All authors contributed to the manuscript revision, read, and approved the submitted version.

## Funding

This work was supported by the Health Committee of Saint Petersburg state assignment for Saint Petersburg Clinical Research and Practical Center of Specialized Types of Medical Care (Oncological).

## Conflict of interest

The authors declare that the research was conducted in the absence of any commercial or financial relationships that could be construed as a potential conflict of interest.

## Publisher’s note

All claims expressed in this article are solely those of the authors and do not necessarily represent those of their affiliated organizations, or those of the publisher, the editors and the reviewers. Any product that may be evaluated in this article, or claim that may be made by its manufacturer, is not guaranteed or endorsed by the publisher.
